# Buffalo Academy of Medicine

**Published:** 1905-03

**Authors:** Franklin W. Barrows


### Buffalo Academy of Medicine.

*Section on Medicine, January 10, 1905.*

Reported by FRANKLIN W. BARROWS, M. D., Secretary.

The regular meeting of the medical section was held Tuesday evening, January 10, at the academy rooms in the Public Library building. The section was called to order at 8.55 o'clock by the chairman, Dr. Allen A. Jones. The minutes of the last meeting were read and approved.

Dr. DeLancey Rochester read a paper entitled,

THE VALUE OF CERTAIN PROCEDURES IN THE TREATMENT OF PNEUMONIA.

*(Abstract)*

The rational mode of treatment falls under these heads: (1) to institute such measures as will relieve toxemia; (2) to prevent failure of heart; (3) to so treat the local conditions in the lung as to retard the progress of congestion to consolidation and hasten the progress of consolidation to resolution; (4) to meet complications as they arise; (5) to prevent the spread of the disease to other individuals.

The "expectant plan" of treatment, in which patient is kept quiet, sponged regularly, fed properly, bowels kept open and complications met promptly as they arise, meets the fourth and fifth indications if sputum is destroyed and stools, urine and bedding disinfected. Its death-rate is from 20 to 40 per cent.

Treatment by large doses of digitalis early in the disease to overcome impending congestion of lung, prevent consolidation and thus jugulate the disease at the start, is said to be very successful. But the majority of these favorable reports seem to be based on doubtful diagnosis and a wrong conception of the nature of the disease.

The Brand treatment of typhoid fever when applied to pneumonia meets few, if any, of the indications to be met. It has not resulted any better than the expectant plan, perhaps not as well.

The treatment by the pneumococcus antitoxin is the natural, rational plan suggested by the infectious nature of pneumonia. So far, it has not always been possible to get a reliable serum, and reports of results vary accordingly. Considering the doubtful efficacy of the serum, its cost is practically prohibitive.

The creasote or guaiacol treatment has been highly lauded. I have been unable to find statistics of this treatment. It seems irrational to add another poison to a patient already profoundly toxic. Creasote tends to increase the inflammation of the kidneys, which usually occurs in pneumonia.

The treatment advocated in this paper is sustained by a record of 201 cases of pneumonia, with a death-rate slightly below 8 per cent. So far as I know, these figures are not equaled by any other plan of treatment.

The first indication in treatment, relief of toxemia, is met by induced sweats and mild catharsis. At the onset of the disease calomel is given, followed by enough of a saturated solution of Epsom salts to produce one or two free evacuations daily. The calomel may be repeated as needed. Sweating is best induced, not by drugs, but by the hot mustard footbath, scientifically given in bed without disturbing the patient.

To prevent further bacterial invasion the mouth is frequently and thoroughly cleansed with a mild antiseptic solution. The diet is fluid and, so far as possible, sterile. Plenty of pure water is administered, in small amounts and often, to flush the excretory organs. The patient is placed in a room having the best possible ventilation and the most direct sunlight. A serious danger in pneumonia is overdistension of the right heart, which, if persisting, always causes death. It can often be prevented by careful study of the lung condition and of the heart and the institution of the treatment described below. A careful physical examination of the chest should be made by the physician at every visit. In this way he can tell when congestion is beginning in any part; and by vigorous local treatment can often prevent further progress of the morbid process. When congestion is beginning in a portion of the lung the prompt and thorough application of leeches, wet cups or dry cups, over the part will often stay the process; or it will delay it twenty-four or forty-eight hours while some previously involved part of the lung is clearing up. Dry cupping should be very thorough, the cups left on for a half-hour and the process repeated every four to six hours. During the intervals the chest should be covered with a light woolen jacket. The poultice jacket should be relegated to the dark ages.

When, in spite of these local measures, the process persists and we begin to see evidence of overdistension of the right heart, —such as cyanosis, pulse losing tension and becoming small and sometimes irregular, enlarged liver, pulsating veins, area of cardiac dulness increasing chiefly to the right,—whether a murmur of tricuspid insufficiency is present or not, prompt relief is usually obtained by withdrawing 8, 10 or 12 ounces of blood from the median vein of the forearm. This may be repeated several times, if need be. It not only relieves the right heart distension, but it withdraws from the body a decided amount of toxic material. At this time a little digitalis, given with due regard to its physiologic effect, may be of decided value. Two cases, illustrating the value of these procedures, are described in detail; one case received 500 to 1000 c.c. of normal salt solution, hypodermatically, once or twice a day until the crisis.

Hypodermoclysis of normal salt solution is now recognised as of the utmost value in the treatment of apparently desperate cases. This is illustrated in four cases of alcoholic pneumonia with delirium tremens and pronounced toxemia, treated during the last year. If more than one lobe is involved, pure oxygen gas has proved of undoubted value in several cases.

While these various procedures are being carried out, direct stimulation of the heart is of the utmost importance; it should be begun as soon as diagnosis is made and not delayed until the appearance of cardiac failure. We should rely upon strychnin, brandy and ammonium carbonate or aromatic spirits of ammonia. Ammonium compounds are usually well borne by the stomach if combined with liquor ammonii acetatis and administered in mucilage or milk. Complications should be met promptly. Pericarditis is best treated, and sometimes cut short, by leeches over the precordium, followed by persistent and continuous application of the ice bag. If, in spite of treatment, fluid accumulates enough to embarrass heart action, it should be promptly evacuated, whether pus or serum.

For acute endocarditis the following out of the general plan of treatment of pneumonia, with enforced rest for some time longer than is usually necessary, with the persistent hypodermic use of nuclein, gives best results. The pain of acute pleurisy is usually best relieved by cupping, with a small hypodermic of morphine when needed. If it goes on to effusion it should be aspirated if serum, and drained if pus. Lung abscess should be promptly evacuated and drained through the chest wall.

If kidney is much involved it is best treated by persistence in fluid diet, milk and mildly alkaline waters in large amounts, by sweats, and leeching or cupping the loins.

Meningitis, not always fatal, is best treated by leeching the back of the neck and the temples, by the ice cap, exclusion of light and noise, and the administering of a combination of antipyrin and sodium bromid which can be continued several days.

Summary: (1) sustain metabolism by easily digested or predigested foods, large amounts of pure water, and oxygen inhalations whenever the absorbing surface of the pulmonary mucosa is so involved as to interfere with proper metabolic oxygenation; (2) sustain elimination (*a*) by the liver and bowel, through the vigorous use of calomel and salts; (*b*) by the skin, through sweats induced by external heat; (*c*) through bleeding when indicated by right heart distension; (3) stimulate heart by strychnin, alcohol or ammonium carbonate, and in suitable cases by the subcutaneous injection of normal salt solution; (4) treat lung locally by leeching, wet or dry cupping, as indicated; (5) recognise complications as they arise and treat promptly; (6) prevent spread of disease by destruction of sputum by burning, and by disinfection of urine and feces, and the boiling of clothing and bedclothes.

That it is a rational attempt to meet the therapeutic indications in the treatment of pneumonia, cannot be denied. That it accomplishes its object better than any other method of treatment in vogue at the present time, is proven by the cases cited.

DISCUSSION.

Dr. G. Tartaro: As an Italian, I am interested in serum therapy. I have treated three cases with the serum prepared by Professor Pane, of Naples. He advises the use of 10 to 15 c.c. each day for four days. Other men have used less of the serum with less resulting benefit. I will cite several cases in which this treatment was followed by improvement in temperature and other symptoms. I will pass around an original package of the Pane serum. I hope the members of the academy will give it a fair trial.

Dr. Van Peyma: We rarely listen to a paper so full of meat as this. Did the author insist on the use of nuclein throughout the course of endocarditis?

Dr. Thoma: Why does not Dr. Rochester use camphor in this disease? If strychnin is used too early in the disease will it not prejudice the chances of recovery?

Dr. Sherman: I am surprised that the author did not mention nitroglycerin as of use in relieving the strain on the heart,—specially to tide over the case until free catharsis is produced. Does not alcohol act as a depressant,—a negative rather than a positive stimulant?

Dr. Blaauw: This is the age of serum therapeusis. In Würzburg the government makes the use of serum obligatory in all cases of pneumococcus infection of the cornea (*ulcus serpens, after Römer*).

Dr. Ullman: The wound made by the leech often bleeds for some time after the removal of the leech; this is due to the effect of the salivary secretion of the leech upon the fibrin of blood. The extract of leeches, which is on the market, ought therefore to have therapeutic value in combating the increased fibrin formation which characterises pneumonia. The benefit of salt solution in pneumonia is suggested by the reduction in the amount of chlorids in the urine. Pericarditis is helped by Credé's ointment and ichthyol.

Dr. Dunham: This is the treatment for pneumonia,—heroic and thorough. It keeps patient and nurse busy all the time.

Dr. Allen Jones: One point in this treatment impresses me as most important,—the absence of antipyretics. The use of these drugs is the chief fault in many plans of treatment.

Dr. Rochester, in closing: The antitoxin treatment is the rational method, provided we can get the right antitoxin. I have used only the American serums, and found them useless. Their price and their doubtful efficacy are enough to exclude them from use at present. The constant use of nuclein works well in combating the infectious process of the endocarditis of pneumonia. I do not think that the early use of strychnin will tire out the heart. I find that camphor frequently nauseates the patient. Its use hypodermatically is so painful that I have not tried it. Nitroglycerin is useful as a capillary dilator, but the hot mustard foot-bath every four to six hours will dilate the capillaries sufficiently. As to alcohol, I use it in the form of brandy or Tokay or some similar wine; in this form the effect of various ethers is combined with that of the alcohol. Although the chlorids are diminished in the urine of pneumonia, yet we do not find in this or in any other fact a sufficient explanation of the rational use of salt solution. The unguentum Credé over the precordium is useful, but leeches usually cut short the pericarditis, if four to eight are used, and the bites are allowed to bleed as long as they will. Experiments demonstrate that a temperature of 104° to 105° kills the pneumococcus. Thus the fever of pneumonia may be beneficial, and, possibly, the use of antipyretics would give the coccus a chance to develop. I would use very few medicines in this disease. Elimination, sweating and local treatment are valuable measures.

Dr. Grover W. Wende read a paper entitled,

### THE PATHOLOGICAL ACTION OF THE ROENTGEN RAYS.

(*Abstract.*)

In this paper all up-to-date literature is carefully examined,—that relating to microscopical findings concerning the devitalising of tissue elements and subsequent degeneration by long exposures and the close application of tubes to the skin. It is established by discussion that the more highly differentiated structures, hair follicles, glands, nails and bloodvessels are more easily affected than the epidermal cells and fibrous stroma; that the cells representing disease are less resistant than the normal cells; and that the normal cell may be stimulated to a healthy process. The views of Baumann and Lenser are given, who dissent from the opinion that the epithelium is the tissue which is most speedily and severely damaged by the rays and maintain that the bloodvessels are the structures primarily and most seriously affected; and they regard the damage done to the bloodvessels as first among the effects produced by *x*-ray.

The bactericidal experiments on cultures of the more common organisms by Freund, Zeit, Wolfinden, Forbes Ross, Dunham and others are quoted. There seems to be a certain degree of unanimity in the opinion that instead of being inhibited, the cultures are stimulated to excessive growth, although the rays may be capable of indirectly interfering with the life of the bacteria in the tissues.

The theory is advanced that the effect is to render the tissues unfit for further growth of the bacteria in them and that this effect is the result of increased phagocytosis. Another phenomenon is advanced as the result of a contributory action of the rays,—that is, a cumulative process, by the action of which, when the cellular degeneration reaches a certain degree, the toxic process in the breaking down of the cells is capable of setting up an inflammatory action.

A short report of the microscopical findings of an *x*-ray burn was given which was similar to those cases reported in previous literature.

DISCUSSION.

Dr. Sydney A. Dunham: We cannot learn the technic of the *x*-rays by reading of it in books. The rays cure most quickly those growths that have a specific organism. They act as a stimulant to the normal activity of the tissues and they cause retrogressive changes in diseased cells.

Dr. A. W. Bayliss: I am glad to see Dr. Wende occupying the ground that he takes in this paper. In view of the extent to which the *x*-rays are now employed it is singular that they are so little understood. We do not know beforehand what action our treatment will have on the patient. Certain anatomical regions are better affected than others. I have had poor results in treating the lip, mouth and tongue. One case of cancer of the lip has gradually grown worse under treatment. In the case of the nose, eye, ear,—the upper parts of the face, in general,—and cervix uteri, treatment has given excellent results. But, in any case, we are unable to promise sure results to the patient.

Dr. Wende, in closing: *X*-ray burns come only from soft tubes used close to the lesion. Every case of superficial epithelioma will yield to the *x*-rays. But this is not true of ulcer. Ringworm and favus do not yield. But sycosis and acne vulgaris are helped more by the *x*-rays than by any other treatment. In ulcers having a hard, cartilaginous margin the inflammatory reaction set up by the *x*-rays is useful. This method is certainly not promising in the treatment of lupus and carcinoma.

Meeting adjourned. Total attendance, 44.

---

Gauze Drains.—Gauze drains left in wounds should not, as a rule, be removed until the fourth day, when the adhesions have liquefied and the gauze is easily removed. In the uterus gauze should never be left in more than twenty-four hours, owing to danger of sepsis.—*International Journal of Surgery.*

